# Endometriosis Surgery during the First Wave of the COVID-19 Pandemic: A Brazilian Single Institution Experience

**DOI:** 10.1155/2021/5040873

**Published:** 2021-10-26

**Authors:** Claudio Peixoto Crispi, Claudio Peixoto Crispi, Eduardo de Almeida Nogueira, Pietro Cardoso Balthar, Camilla Gabriely Souza Guerra, Marlon de Freitas Fonseca

**Affiliations:** ^1^Crispi Institute of Minimally Invasive Surgery, Rio de Janeiro, RJ, Brazil; ^2^Anestesia Carioca Serviços Médicos, Rio de Janeiro, RJ, Brazil; ^3^Department of Women's Health-Fernandes Figueira National Institute for Women, Children and Youth Health-Oswaldo Cruz Foundation, Rio de Janeiro, RJ, Brazil

## Abstract

*Introduction*. Early in the 2020 Coronavirus pandemic stay-at-home guidelines, there were public health orders that elective surgeries be deferred to prioritize hospital beds for critically ill COVID-19 patients. Besides, several reasons led to the postponement of consultations, diagnostic tests, and elective therapeutic procedures. As a result, some women with endometriosis faced chronification of their pain and decreased prospects for pregnancy. The aim of this study was to describe individual responses to minimally invasive complete excision of endometriosis through 40 days of follow-up of women whose endometriosis was considered severe enough to proceed with surgery during the fourth, fifth, and sixth months of constraints imposed by the pandemic. Preventive strategies and safety measures employed to protect patients and staff from acquiring or transmitting Coronavirus infection are presented. *Case Presentation*. This case series report enrolled 11 consecutive Brazilian women (ages 22 to 47 y) who underwent minimally invasive surgical treatment of endometriosis between June 26 and August 17, 2020. Cases of endometriosis requiring more urgent surgery were promptly identified and considered individually. The strict safety measures were well accepted by patients. No women developed any flu-like or COVID-19-related symptoms (cough, dyspnea, fever, or anosmia) in the 40 days of postoperative follow-up. One of the most praised measures reported by patients was the routine testing of the patient, the person who would accompany her in the hospital, and all medical staff and employees. *Discussion*. It is feasible to safely perform elective endometriosis surgery in selected cases during a pandemic.

## 1. Introduction

The spread of the highly contagious respiratory disease—later named COVID-19 by the World Health Organization—caused by the novel Coronavirus (SARS-CoV-2) became evident in Brazil in February 2020, after an individual who had returned from a trip to Italy tested positive for the virus. As with many countries around the world, the news about the rapid spread of the Coronavirus and its significant mortality sowed concern and fear in Brazil. By mid-March 2020, governors of several Brazilian states had ordered public and private schools, cinemas, theatres, and stadiums closed. Federal and state health officials issued recommendations to slow transmission and to prepare public and private health systems and institutions for the expected onslaught of patients requiring hospitalization and intensive care.

As the number of COVID-19 cases and hospitalizations grew exponentially in a matter of weeks, public health officials ordered or recommended the suspension and postponement of elective procedures and surgeries, both to reduce the potential nosocomial exposure of noninfected patients to the Coronavirus and to preserve hospital beds and intensive care unit (ICU) resources for the expected demand as the incidence of Coronavirus cases grew. At the same time, many surgeons and patients chose to cancel or postpone elective procedures out of an abundance of concern about the risk of contracting the Coronavirus in healthcare facilities or simply to comply with “stay-at-home” orders [1]. These policies and recommendations and choices by providers and patients promptly led to the deferral of a significant portion of gynecological procedures in Brazil, including minimally invasive surgical procedures for the management of endometriosis.

Endometriosis is an endemic condition that is associated with pain and different dysfunctions [2–5]. Cytoreductive surgery is the treatment of choice to improve health-related quality of life in cases in which medical management has been ineffective for pain relief or in selected cases of endometriosis-related infertility [6–8]. Extensive resections may be necessary when multiple deep infiltrating lesions occur [3]. Even into the first weeks of 2021, many women with endometriosis have postponed consultations, diagnostic tests, and elective therapeutic procedures, thus allowing the disease to follow its natural course with chronification of pain and decreased likelihood of pregnancy.

As our understanding of how the virus is transmitted and what protective equipment and procedures best protect healthcare workers and uninfected patients evolved in 2020, gynecologists at some hospitals and ambulatory surgical centers, including those at our institute, began to address the backlog of medically indicated procedures and surgeries [9]. The aim of this study is to share our experience with cases of women who presented in 2020 with endometriosis that was considered severe enough to warrant undergoing surgery, beginning five months after “social distancing” measures were instituted. We describe the preventive strategy and sanitary and safety measures that we used to protect staff and patients. This case series reports and discusses individual responses to minimally invasive, nerve-sparing, complete excision of endometriosis at follow-up up to six weeks after surgery.

## 2. Case Presentation

### 2.1. Study Design

This preplanned interdisciplinary observational study enrolled women who were referred by their personal gynecologist to the Crispi Institute for Minimally Invasive Surgery (Rio de Janeiro, RJ, Brazil) for consideration of minimally invasive cytoreductive surgical treatment of endometriosis for infertility and/or pain persisting after medical management. The preoperative assessments were conducted on an outpatient basis, and the recommendation for surgery was made at the discretion of the Institute's attending gynecologist. The surgeries were performed at different hospitals in Rio de Janeiro, which had adopted strict and comprehensive prevention measures against COVID-19 transmission as detailed below. The number of surgeries performed in this period was modest (11 cases) as less urgent cases were deferred and some women simply opted to wait.

Written prospective consent for inclusion in observational studies was signed prior to surgery by all patients involved in this study, who gave their written informed consent for the publication of details of their cases in this article. The 11 cases included in this study constitute a subgroup of a larger research protocol, which evaluates the follow-up of endometriosis surgeries. The research protocol was approved on February 21, 2019, by an institutional review board—the Research Ethics Committee of the Oswaldo Cruz Institute Foundation (CAAE 07885019.8.0000.5269 IFF-FIOCRUZ), which authorized the inclusion of patients admitted to the Crispi Institute for Minimally Invasive Surgery since January 2018. Patients who might have declined to take part in the study would have received the same care as the patients who gave their consent to take part in the study. Demographic, clinical, and outcome data were abstracted from the medical records into a database in December 2020.

Both the Strengthening the Reporting of Observational Studies in Epidemiology (STROBE) [10] statement and the updated Preferred Reporting of Case Series in Surgery (PROCESS) [11] guidelines were followed to improve the quality of reporting.

### 2.2. Operative Procedure

Our multidisciplinary endometriosis referral center manages the diagnosis and treatment of endometriosis following the guidelines of the American Society of Reproductive Medicine [12] and the European Society of Human Reproduction and Embryology [13]. The diagnosis of endometriosis involved four steps: (1) medical history, (2) physical examination, (3) magnetic resonance imaging (MRI), and (4) histological confirmation after laparoscopy.

In this case series, an experienced multidisciplinary team led by the same gynecologist (C.P.C.) performed all surgeries using a nerve-sparing cytoreductive surgical strategy for complete excision of the endometriotic lesions. During laparoscopic exploration of the abdominal cavity—whether robot-assisted or not—the lesions previously identified by physical examination and MRI were assessed and resected. In some cases, surgery incorporated transoperative cystoscopy to address complex bladder or ureter lesions and hysteroscopy to address intrauterine conditions.

The Foley catheter was removed from the bladder once the residual urine volume was consistently <100 mL. After discharge, the patients were followed by the multidisciplinary team (gynecologist, proctologist, urologist, psychologist, and nutritionist) for a minimum of 40 days. Endometriosis was confirmed histologically in all 11 patients.

### 2.3. Specific Safety Considerations to Schedule the Surgery

During the period of this case series, only surgeries considered more urgent in the view of both the responsible surgeon and the patient were scheduled. Although the imperative to operate during the Coronavirus pandemic varied with each woman according to the indication for surgery and the particularities of each case, the safety considerations and rules were more uniform: (1) elective surgeries should only be performed in facilities considered “COVID-free” (such facilities do not care for patients with COVID-19 and routinely test health professionals and staff for Coronavirus with RT-PCR tests of nasopharyngeal swabs every 7 or 14 days); (2) both the patient and a single companion (who could stay in the patient's room) were tested for Coronavirus 3 or 4 days prior to surgery; (3) the patient and family members received guidelines for social isolation, especially during the two weeks prior to surgery; (4) the patient should be accommodated in a private room under a contact isolation regime to prevent cross-infection; (5) nursing staff should have used appropriate personal protection equipment (PPE) including disposable gowns, N95 masks (or similar), protective goggles, and gloves; and (6) no external visits or companion exchange was allowed during the hospitalization.

### 2.4. Specific Anesthesia Safety Considerations

Two experienced anesthesiologists (E.A.N. and P.C.B.)—each who has worked with this team for more than 10 years—participated in these 11 surgeries. The anesthetic strategy was the same used before the Coronavirus pandemic—a combined regional-general method. First, spinal anesthesia was performed with isobaric bupivacaine and morphine. General anesthesia was then induced with intravenous propofol, alfentanil, and rocuronium bromide (nonrapid sequence of induction and intubation) and maintained with inhalation sevoflurane. Special attention was paid during orotracheal intubation and during extubation; appropriate PPE was used systematically.

### 2.5. Specific Pneumoperitoneum Safety Considerations

**Figure 1:**
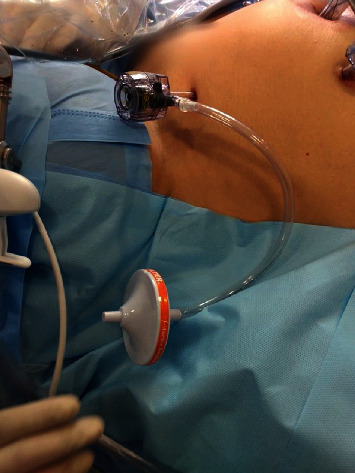
HEPA filter systematically used in the exhaust of the artificial pneumoperitoneum and smoke produced.

With a focus especially on some known risks of contamination of the operating theater staff during surgery, the use of PPE was not considered sufficient and some specific measures were also adopted to minimize the dispersion of low-temperature aerosols. To prevent accidental loosening during surgery with consequent gas escape, disposable trocars with grooves were systematically preferred and carefully fixed by suturing with 3.0 nylon. Efficient air filters (such as the HEPA filter—Figure 1) were systematically used in the exhaust of the artificial pneumoperitoneum and smoke produced. The gas from the abdominal cavity was always exhausted “smoothly” and as much as possible before removal of specimens (in bags), which were handled carefully and transferred to the pathology department. Finally, residual CO_2_ was evacuated from the abdominal cavity before removing the trocars.

Uterine manipulators are frequently used in simple and radical hysterectomies and are beneficial in improving visualization and providing a landmark for the colpotomy. In this series, whenever a colpectomy/colpotomy was necessary, a uterine manipulator with a vaginal occluder balloon was used. The balloon was filled just before the opening of the vaginal mucosa. Mechanical occlusion of the distal part of the vagina and the vulva with a humid compress was also performed to prevent gas leakage.

### 2.6. Specific Safety Considerations to Protect the Patient

Basically, the strategy to minimize the risk of infection by Coronavirus during the hospitalization was to bar visits (only a single companion could accompany the patient) and to promote the consistent use of masks and frequent hand hygiene. No “prophylactic drug” was used.

After extubation, patients remained in the operating room under the care of the anesthesiologist until they were able to be taken directly to their room bypassing the Recovery Room. This avoided being in close proximity to other patients. Without compromising appropriate care, the postoperative clinical management was sought to discharge the patient to home as early as possible. All the patients were instructed to maintain social isolation after surgery.

During multidisciplinary follow-up, telemedicine consultations were favored whenever possible and an active search for symptoms of Coronavirus infection was made during each “encounter” whether by telephone, by video call, or in person. As no specific signs or symptoms of COVID-19 emerged in any of the 11 patients in this series during the first 40 days after surgery, no specific viral or antibody testing was ordered.

The anthropometric characteristics, obstetric histories, and principal complaints related to endometriosis (which constitute the indication for surgery) are presented for each of the 11 cases in Table 1.

The strict safety measures were well accepted by all patients. No patient developed any “flu-like” symptoms or other symptoms associated with COVID-19 (cough, dyspnea, fever, or anosmia) in the 40 days of postoperative follow-up. Although two patients reported fear of contracting the COVID-19 during hospitalization, all women thought that the preventive and safety measures adopted by the hospital and the team were “adequate” (7 women) or “more than adequate” (4 women). One of the most praised measures was the preadmission RT-PCR testing of the patient and her companion and the routine testing of medical staff and employees.

Of the 11 patients, one (case #2) reported having been diagnosed with COVID-19 two months prior to her surgery. She had a positive IgG serology. Nevertheless, she and her companion underwent a PCR exam prior to surgery; both were negative. In another case (case #8), the patient's companion (husband) tested positive. Even though both were asymptomatic and the patient tested negative for Coronavirus, her surgery was postponed for 14 days.

During the period described in this series, one of the physicians of our team tested positive for Coronavirus. He was asymptomatic at the time of specimen collection. He self-isolated for 14 days and did not develop symptoms.

In this series, no procedure was converted to open surgery. A few hospitalizations were longer than predicted. In case #5, there was a complication (bowel anastomosis leakage) inherent to the extent of her endometriosis and the complexity of the surgery. The details of the surgeries for the treatment of endometriosis at a “COVID-free” facility following specific Coronavirus infection prevention protocols are presented in Tables 2 and 3.

## 3. Discussion

This case series study presents short-term clinical outcomes (up to 40 days) of minimally invasive surgeries for deep infiltrating endometriosis performed during a period characterized by extreme concern about the risks inherent to the COVID-19 pandemic. In addition to describing some anatomical details of each surgery performed—including the location of the endometriosis lesions that were identified and treated—our report also highlights specific safety measures adopted to prevent the healthcare team and patients from becoming infected by this novel Coronavirus. In this series, each patient and our team engaged in discussion and together weighed the risk of acquiring COVID-19 against the benefits of the surgery.

Decisions early in the pandemic to postpone elective operations for benign gynecologic diseases were considered imperative to reduce virus transmission among the population and to preserve healthcare resources for an expected influx of COVID-19 patients and for surgeries that could not be safely deferred [14]. The Brazilian Federal Medical Board (*Conselho Federal de Medicina*) promulgated rules and recommendations—initially valid for a period of three months—against performing elective surgeries in the public and private health systems. These policies generated considerable concern and were not renewed in the original form.

Despite the evidences that deep infiltrative endometriosis surgery improves pain [15] and has a positive impact on evacuation symptoms and bowel function [16], the treatment of endometriosis and infertility prompt complex clinical questions without simple answers. It is important to acknowledge that for many women, life goes on and the burden of chronic disease still needs to be managed, even if resources are being diverted to other areas in the midst of a global focus on COVID-19 [17]. In contrast to the inherent uncertainties in dealing with a novel and poorly understood infection such as the SARS-CoV-2 Coronavirus and its clinical manifestations (COVID-19), endometriosis is a relatively well-understood condition, which tends to progress over time despite hormonal blockade. In other words, time is an important factor for many women.

As healthcare providers treating chronic pelvic pain patients (a vulnerable population), we need to adapt with them and offer all the tools available to complement their treatment [18]. Many women suffering from pelvic pain have delayed seeking treatment. Given the likelihood that this pandemic will continue for many months ahead, we must keep an open mind [19]. Yet, the impact of the COVID-19 pandemic on the infertile couples who should have undergone in vitro fertilization (IVF) treatment has been significant. In many communities in Brazil, the abrupt suspension of IVF cycles is one of the many healthcare services postponed to enable the reallocation of staff and resources to deal with the COVID-19 pandemic [20]. Within available resources, dedicated “COVID-19-free” facilities should be established during current and before future SARS-CoV-2 outbreaks, which are still expected despite the introduction of the various vaccines [21].

Although there seems to be no reason to abandon laparoscopic surgery in favor of open surgery, the risks should not be underestimated. Surgery should be performed on patients with Coronavirus only when necessary and healthcare providers should use logic and common sense to protect themselves and others by performing surgery in a safe and protected environment [22].

The reality that surgeons and anesthesiologists often work in more than one institution or facility makes it imperative that these health professionals be tested for Coronavirus at appropriate intervals. However, now that most health professionals have been vaccinated against the Coronavirus, whether and how such systematic testing should be continued needs to be revisited and updated, mainly, with the expectation of an overload of surgical procedures to come that were postponed previously [23].

Regarding the limitations of the study, we highlight the limited number of cases, which makes it impossible to estimate the risk not only of surgical complications but also of infectious complications (including COVID-19). Postoperative testing for the Coronavirus was not systematically ordered after surgery because no patients reported COVID-19 signs or symptoms, and the utility of such testing likely would have been extremely limited. Thus, our study did not pursue asymptomatic Coronavirus infection during the six weeks after surgery.

Several questions regarding the transmissibility of the COVID-19 remain unanswered [9]. During a pandemic period characterized by confused information, nonconsensual opinions, and scientific uncertainties [24], cases of endometriosis requiring more urgent surgery should be promptly identified and the benefits weighed against the risks on a case by case basis. In addition to carefully assessing the degree to which severe symptoms, secondary infertility at an advanced age, or the risk of organ dysfunction create a sense of urgency, it is extremely important to alert each patient not only about the risks of surgical complications or of contracting COVID-19 but also outline the range of strategies currently available to perform an elective (“nonemergency”) surgery of high complexity with both comfort and safety [25].

The COVID-19 pandemic has changed working conditions for surgical teams around the world, including the restructuring of surgical schedules, staff preparation, and the department outbreak response protocols and recommendations for surgical techniques and risk management [26, 27]. In both emergency situations and elective procedures, the risks and benefits of laparoscopy and open surgery should be considered and discussed with each patient [28, 29]. Regarding the general precautions and strategies for the staff to overcome transmission during clinical practice, surgeons may perceive some impediment for both visibility and communication; their perceived lack of protection and comfort and increased fatigue may inhibit their optimal surgical performance [30]. As already stated, the postponement of an endometriosis surgery may allow the disease to follow its natural course with chronification of pain and decreased likelihood of pregnancy. Lessons learned from healthcare professionals who have managed high volumes of surgical patients during the pandemic could be useful to mitigate some risks and reduce exposure to other patients and public and healthcare staff [31]. The option of telemedicine for outpatient follow-up after endometriosis surgery instead of an outpatient clinic proved to be quite adequate—similarly to what has been proposed for a follow-up of conservatively treated appendicitis with antibiotics during the COVID-19 pandemic [32]. In this series, telemedicine consultations were favored whenever possible during multidisciplinary follow-up.

In conclusion, selected cases of endometriosis in which postponing surgery would be prejudicial to the health of the woman or her reproductive prospects should be carefully considered because it is feasible to safely perform elective surgery during a pandemic.

## Figures and Tables

**Table 1:** Anthropometric characteristics, obstetric histories, and indication for each surgery.

Case	Age (years)	Ethnicity	Schooling	Weight (kg)	Height (m)	BMI (kg·m^−2^)	G P A	Menarche (years)	Indication
1	45	Caucasian	Postgrad	60	1.55	24.97	1 1 0	11	Pain
2	37	African	College	62	1.72	20.96	1 1 0	12	Pain
3	38	Caucasian	Postgrad	56	1.57	22.72	4 1 3	12	Pain
4	38	Caucasian	Postgrad	64	1.65	23.51	0 0 0	13	Pain
5	36	Caucasian	College	56	1.56	23.01	0 0 0	13	Pain
6	31	Mixed	College	75	1.64	27.89	0 0 0	10	Pain
7	22	Caucasian	High school	65	1.6	25.39	0 0 0	10	Pain
8	47	African	High school	65	1.57	26.37	3 1 2	12	Pain
9	31	Caucasian	College	71	1.74	23.45	0 0 0	14	Pain
10	38	Caucasian	Middle school	67	1.6	26.17	3 1 2	14	Infertility
11	31	Caucasian	College	70	1.58	28.04	0 0 0	13	Imaging

Cases ordered according to the date of surgery. Ethnicity: self-reported; schooling: highest degree completed; G P A: obstetric history includes G (gravidity, number of times a woman has been pregnant), P (parity, number of times a woman carried the pregnancies to a viable gestational age), and A (abortion, number of abortions—spontaneous or not); indication: in all cases, women underwent surgery due to pain, except for case 10 (secondary infertility) and case 11 (risk for bowel obstruction and concerns about imaging findings showing endometriosis invading the appendix, the ovaries, and the anterior, posterior, and lateral compartments). All women are nonsmokers.

**Table 2:** Details of the surgeries.

Case	Surgery	Duration (min)	Bleeding (mL)	Discharge (days)	Foley (days)	Obs.
1	Robotic	164	30	1	1	Horizontal continuous colporrhaphy was performed
2	Laparoscopy	180	60	2	1	Increased surgery time due to equipment issues
3	Laparoscopy	320	70	3	1	Abdominal wall endometriosis; punctures directly on the abdominal musculature
4	Robotic	105	20	1	1	—
5	Robotic	550	500	18	18	Reoperation (ileostomy) due to anastomosis dehiscence + rectovaginal fistula
6	Laparoscopy	490	400	6	21	Parametric involvement reaching the pelvic floor bilaterally
7	Laparoscopy	103	50	2	1	—
8	Laparoscopy	302	200	3	20	Left nephrectomy + internal iliac chain lymphadenectomy to reach deep lateral compartment
9	Robotic	135	30	3	1	Bilateral lymphadenectomy of iliac chains and right obturator fossa
10	Laparoscopy	200	100	3	1	Complex myomectomy and adenomyomectomy
11	Laparoscopy	140	20	2	1	—

Cases ordered according to the date of surgery. Foley: time to remove the Foley catheter; case 7: the bowel anastomosis leakage was diagnosed and treated surgically during the same hospital stay. The option for robotic-assisted surgery in some cases was unrelated to protective measures against COVID-19.

**Table 3:** Systematic description of surgical procedures performed at the main sites affected by endometriosis.

Case	Tube	Ovary	Uterus	RLig	Bladder	Ureter	Param	Nerve	UsLig	Vag	Rcerv	RSep	Bowel
	(R/L)	(R/L)		(R/L)		(R/L)	(R/L)		(R/L)				
1	H + S/H + S	/F	A	X/X	V + P		X/		X/X	C	X	X0	rS
2	B/P	F/PEF	B		V + P	/E	Xd/		X/X	S	X	X0	pS
3	B/P	F/F	I		P				X/X		X		pS + rS + Sut
4	P/P	F/PF	M		V + P		Xd/		X/X		X	X0	
5	P/P	PFE/F	P	X/X	S + V	E/	Xd/Xd	#1	X/X	C	X	X	R
6	P + Sp/P	PF/F	P		S + V		Xd/Xd	#2	X/X	C	X	X	R + A
7	P/B + Sp	F/PEF			V + P	/E	Xd/Xd		X/X	S	X	X0	pS + A
8	S/S	/TE	T	X/X		/U	X/Xd	#3	X/X	C	X	X	DD + A
9	P/P	F/F			V + P			#4	X/X				
10	H + S/H + S	F/	A + M + U						X/X				
11	P/P	F/F	A	/X	V + P		Xd/		X/X	S	X	X0	A + pS + D

R/L: right/left. X: excision. Blank space: no endometriosis lesion identified; tube: P pervious or B blocked at chromopertubation; Sp salpingoplasty; S salpingectomy; H hydrosalpinx. Ovary: T oophorectomy; P oophoroplasty; E endometrioma; F presence of endometriosis in the peritoneum of the ovarian fossa. Uterus: M myomectomy; A adenomyomectomy; T trachelectomy (previous subtotal hysterectomy); U bilateral permanent uterine arteries ligation; P hysteroscopic polypectomy; B hysteroscopic biopsy; I hysteroscopic repair of isthmocele. RLig: round ligament excision. Bladder: Sut partial cystectomy and intracorporeal suturing; P superficial nodule infiltrating bladder peritoneum shaving; V endometriotic nodule excision on the vesicouterine septum. Ureter: U ureterectomy (surgical excision of part of a ureter); E extrinsic ureteral endometriosis treated by ureterolysis (systematic bilateral ureterolysis was performed in all cases (procedure aimed at exposing the ureter in order to free it from external pressure or adhesions or to avoid injury to it during surgery)). Param (parametrium): Xd means deeper resection, below the ureter (paracolpium). Nerve (excision of endometriosis nodule infiltrating pelvic nerve): #1 endometriotic nodule resection and decompression of right sciatic nerve, lumbosacral trunk and sacral roots+lymphadenectomy; #2 left hypogastric nerve shaving; #3 partial resection of the distal left hypogastric nerve at the level of the paracolpium; #4 endometriotic nodule resection and decompression of right sciatic nerve, lumbosacral trunk and sacral roots+lymphadenectomy (a nerve-sparing approach including dissection and isolation of hypogastric nerves was used in all cases). UsLig: uterosacral ligament excision. Vag (Vagina): C superior colpectomy+colporrhaphy; S endometriotic nodule excision only (shaving; no suture required). Rcerv: retrocervical area. RSep (rectovaginal septum): X0 means dissected, but without endometriosis. Bowel: A appendicectomy; R colorectal segmental resection; rS rectal shaving; pS perirectal fat shaving; Sut reinforcement suture; D discoid resection; DD double discoid resection. Cases ordered by date of surgery.

## Data Availability

All data used to support the findings of this study are included within the article.
